# Predominant Merkel Cell Polyomavirus DNA Detection in Essential Thrombocythemia within Myeloproliferative Neoplasms

**DOI:** 10.1158/2767-9764.CRC-25-0471

**Published:** 2026-04-03

**Authors:** Dan Liu, Sixuan J. Wang, Amanda Macamo, Kim Severens, Myrurgia Abdul-Hamid, Véronique Winnepenninckx, Mathie P.G. Leers, Axel zur Hausen

**Affiliations:** 1 Department of Pathology, GROW-School for Oncology and Developmental Biology, Maastricht University Medical Centre+, Maastricht, the Netherlands.; 2 Department of Hematology, The Affiliated Hospital of Southwest Medical University, Luzhou, China.; 3 Department of Clinical Chemistry and Hematology, Zuyderland Medical Center, Sittard-Geleen/Heerlen, the Netherlands.; 4 Central Diagnostic Laboratory, School of Nutrition and Translational Research in Metabolism (NUTRIM), Maastricht University Medical Centre+, Maastricht, the Netherlands.; 5 Department of Environmental Sciences, Faculty of Science, Open Universiteit, Heerlen, the Netherlands.

## Abstract

Acute thrombocythemic myeloproliferative disease in mice has been reported upon introduction of middle T gene expression of mouse polyomavirus. Merkel cell polyomavirus (MCPyV) is an oncogenic human polyomavirus that accounts for approximately 80% of all Merkel cell carcinomas. In this study, we assessed the presence of MCPyV DNA in fresh bone marrow (BM) aspirates from patients with myeloproliferative neoplasms (MPN) using MCPyV-specific DNA polymerase chain reaction. MCPyV DNA prevalence was significantly higher in 78 BM samples from patients with MPNs (17.9%, 14/78) than in 66 BM controls undergoing femoral head replacement surgery (3%, 2/66; Fisher exact test, *P* = 0.0063; OR = 7.95% confidence interval = 1.53–32.06). Notably, positivity was predominant in essential thrombocythemia (ET; 11/14). MCPyV mRNA was detected in MCPyV DNA–positive samples, indicating low-level viral transcription. Interestingly, MCPyV positivity was significantly correlated with female sex but not with age or specific MPN genetic mutations, except for myeloproliferative leukemia virus oncogene mutations. These findings suggest a potential association between MCPyV and MPNs, particularly ET, and support further investigation into the role of human polyomavirus in megakaryocytic lineage biology.

**Significance:**

MCPyV DNA was detected in BM samples from patients with MPNs, especially those with ET. This observation suggests possible involvement of MCPyV in megakaryocytic lineage biology, providing new insights into MPN pathogenesis and promoting further investigation into the role of human polyomaviruses in hematologic disorders.

## Introduction

Classic myeloproliferative neoplasms (MPN) include polycythemia vera (PV), essential thrombocythemia (ET), and primary myelofibrosis (PMF). These hematologic disorders are characterized by the excessive proliferation of terminally differentiated myeloid blood cells (1). Although driver mutations in Janus kinase 2 (JAK2), calreticulin, and myeloproliferative leukemia virus oncogene (MPL) have been identified in most cases and active JAK2–STAT pathway, these alterations alone do not fully explain MPN initiation or disease heterogeneity (2).

The recently identified Merkel cell polyomavirus (MCPyV) is a novel human DNA tumor virus. MCPyV is the causative agent of most highly malignant Merkel cell carcinomas (MCC), a rare but very aggressive nonmelanoma skin cancer (3, 4). Notably, it has been demonstrated that MCPyV infects both lymphocytes and monocytic cells (3, 5–9). Furthermore, MCPyV DNA sequences have been reported in various blood-derived specimens from blood donors, including buffy coats, serum, and whole blood (10–13).

Rare reports of aggressive MCC arising in PV patients treated with the JAK2 inhibitor ruxolitinib raise the possibility of a link between MCPyV and MPN biology (14, 15). In experimental models, mouse polyomavirus middle T antigen drives megakayocytic hyperplasia and an acute thrombocythemic disorder, further supporting a potential interaction with the megakaryocytic lineage (16). Despite these observations, the presence of MCPyV in human bone marrow (BM) from patients with MPNs has never been systematically investigated.

In this study, we examined freshly obtained BM samples from patients with MPN and control samples to determine the presence of MCPyV. To our knowledge, this is the first study to evaluate the presence of MCPyV in a unique collection of fresh BM specimens.

## Materials and Methods

### Patients and samples

A total of 78 BM specimens were obtained from patients diagnosed with MPNs, including ET (*n* = 44), PV (*n* = 6), PMF (*n* = 3), Post-ET-MF (*n* = 5), Post-PV-MF (*n* = 3), and chronic myeloid leukemia (CML; *n* = 17; Supplementary Table S1). The median age of the MPN cohort was 67.5 years (range, 29–87), comprising 45 males (57.7%) and 33 females (42.3%; Supplementary Table S2). Additionally, control samples (*n* = 66) were obtained from patients undergoing femoral head replacement surgery at Zuyderland Medical Center (Sittard, Netherlands). Individual demographic information (age and sex) of the control group was not available because of sample deidentification. The inclusion criteria were based on the availability of DNA from fresh BM specimens. The exclusion criteria were based on the corresponding histopathologic diagnosis; thus, attrition was not applicable for this study. The samples were collected following approval from the local ethics committee (METC Z registration no. 15-N-201). The Medical Ethics Review Committee approved the study of Maastricht UMC+ in the Netherlands (2019-0977). To validate MCPyV-large T antigen expression, three MCPyV-negative polymerase chain reaction (PCR) ET patients from paraffin-embedded BM biopsy specimens were obtained from the Maastricht University Medical Center+ (MUMC+) tissue archive (Supplementary Table S3).

### DNA extraction

Genomic DNA (gDNA) was extracted from 3 mL of each BM sample using the ReliaPrep Blood gDNA Kit (Promega, cat. #A5081) following the manufacturer’s instructions. DNA concentration was measured using a NanoDrop2000 spectrophotometer (Thermo Fisher Scientific), and quality was evaluated using specimen control size (SCS) ladders, as previously described (17).

### PCR

PCR amplification was conducted using 250 ng of gDNA extracted from the BM. gDNA from the MCPyV-positive cell line WaGa (RRID: CVCL_E998) served as a positive control for MCPyV, a laboratory-constructed HPyV6 plasmid with His-tag was used as a positive control for HPyV6, and an equivalent volume of H_2_O was used as a negative control. The reaction was performed with AmpliTaq Gold DNA Polymerase (Applied Biosystems, Thermo Fisher Scientific, cat. #4311816) in a 50 μL reaction. MCPyV and HPyV6 genes were amplified with the following primer sets: MCPyV_LT3 forward primer (5′-TTG​TCT​CGC​CAG​CAT​TGT​AG-3′) and reverse primer (5′-ATATAGGGGCCT CGTCAACC-3′); MCPyV_M1/M2 forward primer (5′-GGCATGCCTGT GAATTAGGA-3′) and reverse primer (5′-TTG​CAG​TAA​TTT​GTA​AGG​GGG​CT-3′); MCPyV _VP1 forward primer (5′-TGG​ATC​TAG​GCC​CTG​ATT​TT-3′) and reverse primer (5′-TTTGCCAGCTT ACAGTGTGG-3′); and HPyV6_sTAg forward primer (5′- ATCAGCTTCCACA GGTAGGC-3′) and reverse primer (5′-TTG​CCT​TCT​CAA​AAA​GGA​GC-3′; ref. 18). Following amplification, PCR products were purified using the NucleoSpin gel and PCR Clean-up Mini Kit (Macherey-Nagel, cat. #740609.250, Düren, Germany) and subsequently analyzed by Sanger sequencing using the BigDye Terminator Kit (Applied Biosystems). Sequencing was performed by capillary electrophoresis at the Clinical Genetics Department, MUMC+. Raw sequence data were examined by comparison with reference sequences (NC_010277.2: Merkel cell polyomavirus isolate R17b, complete genome, and GCF_000888495.1: human polyomavirus 6 isolate 607a, complete genome) using the NCBI nucleotide BLAST tool (RRID: SCR_004870). Only samples with sequences aligned with the MCPyV or HPyV6 reference genes were considered positive.

### RNA *in situ* hybridization

The RNAscope 2.5 RED assay kit (Advanced Cell Diagnostics, cat. #322360) and specific RNA probes were used to detect MCPyV and HPyV6 mRNA according to the manufacturer’s instructions (19). Fourteen pairs of probes targeting nucleotides 197 to 1,448 of MCPyV (V-MCPyV-LT-ST-Ag; accession number: NC010277.1) and 20 pairs of probes targeting nucleotides 3,786 to 4,898 of HPyV6 (V-polyomavirus-HPyV6; accession number: HM011563.1) were used. Briefly, formalin-fixed, paraffin-embedded block sections were deparaffinized and pretreated with hydrogen peroxide for 10 minutes, followed by antigen retrieval using target retrieval reagents at 100°C for 15 minutes, and treated for 30 minutes with RNAscope Protease Plus. *In situ* hybridization was performed using the target probe followed by six signal amplification steps. Amplified signals were visualized using Fast Red chromogen, followed by hematoxylin counterstaining. Slides were scanned and images were exported using CaseViewer (RRID: SCR_017654). All RNA *in situ* hybridization (RISH) slides were scored according to the RNAscope signal quantification guidelines (20) using the following grading system: 0, no staining or <1 dot per 10 cells; 1+, 1 to 3 dots per cell; 2+, 4 to 10 dots per cell with very few clusters; 3+, >10 dots per cell, <10% of cells showing dot clusters; and 4+, >10 dots per cell, >10% of cells showing dot clusters. A probe for the housekeeping gene peptidylprolyl isomerase B (PPIB) mRNA expression was used as a positive sample quality control, and a probe for dihydrodipicolinate reductase gene (DapB) expression was used as a negative sample quality control. The MCPyV-negative cell line MCC26 (RRID: CVCL_2585) and MCPyV-positive cell line WaGa were used as negative and positive controls for MCPyV. Laboratory constructed HEK293-HPyV6 early region (ER) and HEK293-Lenti-empty vector cells, derived from parental HEK293 cells (RRID: CVCL_0045), were used as the positive and negative controls for HPyV6.

### Immunohistochemistry

Antibodies against MCPyV-LTAg we used (clone: CM2B4; Santa Cruz Biotechnology, cat. #sc-136172, RRID: AB_2023156). Immunohistochemistry (IHC) staining was performed on a Dako Autostainer Link 48 using the EnVision-FLEX visualization kit (K8008; DAKO) according to standard protocols (21). Briefly, antigen retrieval was performed using a sodium citrate solution. Endogenous peroxidase was blocked with Dako REAL Peroxidase-Blocking Solution, followed by 20 minutes of incubation with the primary antibody. The binding of the antibodies was visualized using horseradish peroxidase and substrate 3, 3′-diaminobenzidine to create a brown precipitate. Hematoxylin was used as a counterstain for the slides. MCPyV-positive MCC tissue was used as a positive control for the CM2B4 antibody. All slides were evaluated by two pathologists (MAH and AzH). Slides were scanned, and images were exported using CaseViewer. The intensity of MCPyV staining was evaluated and scored as positive brown nuclei very strong (++++), strong (+++), moderate (++), and weak (+) and was seen at 40× magnification.

### Data visualization and statistical analysis

Plots were generated using Python (RRID: SCR_008394) in Jupyter Notebook (Project Jupyter, RRID: SCR_018315), utilizing libraries such as Matplotlib (RRID: SCR_008624; ref. 22), seaborn (RRID: SCR_018132; ref. 23), and pandas (RRID: SCR_018214; ref. 24). The clinicopathologic characteristics, including age, sex, diagnosis, mutational status, and peripheral blood count, were analyzed to assess their potential association with the presence of MCPyV. Numerical data were analyzed using the independent *t* test, whereas categorical data were evaluated using the independent *χ*^2^ test in Python. In cases in which the expected frequency was less than five, Fisher exact test was applied. Odds ratios (OR) and 95% confidence intervals (CI) were calculated using the Haldane–Anscombe correction for contingency tables containing zero counts. Statistical significance was determined using a critical *P* value ≤ 0.05.

## Results

We analyzed 78 BM samples from patients with MPN, including ET (56.4%), PV (7.7%), PMF (3.85%), post-ET-MF (6.4%), post-PV-MF (3.85%), and CML (21.8%; Supplementary Table S1). Clinicopathologic data, including mutational status, are summarized in Table 1.

**Table 1.** Clinicopathologic characteristics of MPNs in association with the prevalence of MCPyV.

​	MCPyV		Total	*P* value
	Positive	Negative		
Clinical characteristics	​	​	​	​
No of patients	14 (17.94%)	64 (82.05%)	78	​
Age (years)	67.3 (53–81)	68 (29–87)	67.5 (29–87)	0.9657^a^
Male	4 (5.12%)	41 (52.56%)	45 (57.69%)	0.0327^b^
Female	10 (12.8%)	23 (29.49%)	33 (42.3%)	
White blood cell counts (10^9^/L)	44.1 (2.5–135.6)	49.83 (4–443.8)	49.23 (2.5–443.8)	0.1944^a^
Red blood cell counts (10^12^/L)	4.38 (2.98–5.65)	4.45 (2.3–7.51)	4.43 (2.3–7.51)	0.9426^a^
Plate counts (10^12^/L)	823.96 (273–1,454)	847.76 (62–3,492)	828.73 (62–3,492)	0.6932^a^
Histopathology	​	​	​	​
ET	11 (14.1%)	33 (42.3%)	44 (56.41%)	​
PV	0	6 (7.69%)	6 (7.69%)	​
PMF	1 (1.28%)	2 (2.56%)	3 (3.85%)	0.1860^c^
CML	1 (1.28%)	16 (20.51%)	17 (21.9%)	​
Post-ET-MF	1 (1.28%)	4 (5.13%)	5 (6.41%)	​
Post-PV-MF	0	3 (3.85%)	3 (3.85%)	​
Genetic mutation	​	​	​	​
Jak2 V617F	9 (3.84%)	36 (46.15%)	45 (57.69%)	1^d^
Jak2 exon12	0	0	0	N.A.
Calreticulin	0	5 (6.41%)	5 (6.41%)	0.5526^d^
MPL	3 (3.85%)	2 (2.56%)	5 (6.41%)	0.0243^d^
BCR-ABL major	1 (1.28%)	15 (19.23%)	16 (20.51%)	0.4098^d^

N.A., not applicable, because contingency table is not 2 × 2 table.

a *t*-test, statistical critics <0.05.

b χ^2^ test, statistical critics <0.05.

c Monte Carlo *χ*^2^ test, statistical critics <0.05.

d Fisher exact test, statistical critics <0.05.

**Figure 1.**
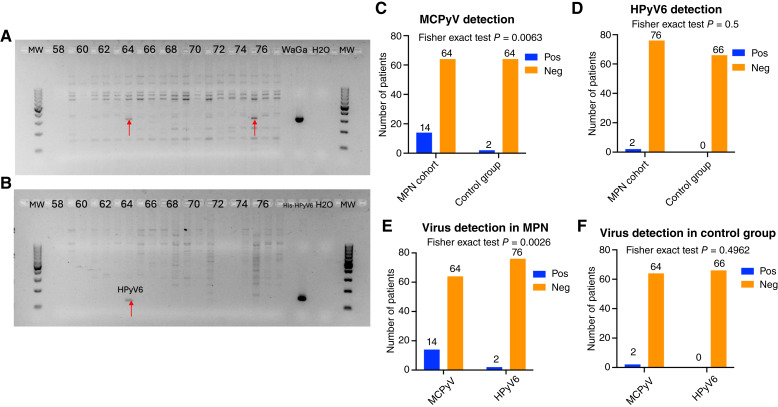
Detection and comparison of MCPyV and HPyV6 DNA in BM samples from patients with MPNs and healthy controls. **A,** Representative gel-electrophoresis of MCPyV DNA-PCR products using LT3 primers, generated from freshly isolated DNA from the BM of patients with MPNs. Specific MCPyV PCR products (confirmed by Sanger sequencing) are indicated by red arrows pointing to the expected 308 bp band. **B,** Representative gel-electrophoresis of HPyV6 DNA-PCR products using sTAg primers, generated from freshly isolated DNA from the BM of patients with MPNs. A red arrow indicates the expected 123 bp PCR product. **C,** Bar plot showing a statistically significant increase in the number of MCPyV-positive DNA samples in the MPN cohort compared with the healthy control group (Fisher exact test: *P* = 0.0063). **D,** Bar plot showing the frequencies HPyV6-positive and -negative samples in the MPN cohort and healthy control group (Fisher exact test: *P* = 0.5). **E,** MCPyV DNA was significantly more frequently detected than HPyV6 DNA in the BM samples of patients with MPNs (Fisher’ exact test: *P* = 0.0026). **F,** No significant difference was observed in the presence of MCPyV DNA and HPyV6 DNA in the control group (Fisher exact test: *P* = 0.4962). *P* < 0.05 as statistically significance. H_2_O, negative control; His-HPyV6, plasmid containing His-tagged HPyV6 genes used as a positive control for HPyV6; MW, molecular weight marker (100 bp); Neg, negative; Pos, positive; WaGa, MCPyV-positive MCC cell line DNA used as a positive control for MCPyV.

**Figure 2.**
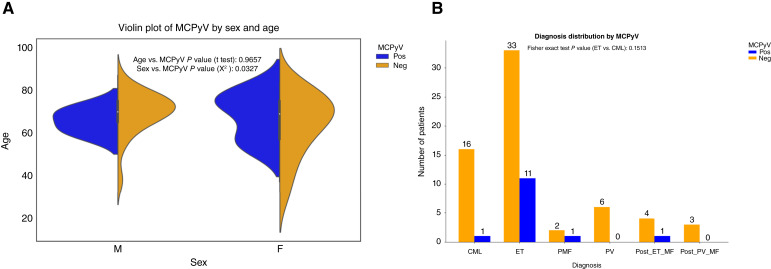
Demographic and diagnostic characteristics of MCPyV-positive and MCPyV-negative patients with MPNs. **A,** Violin plots showing the age distribution across sexes in MCPyV-positive (blue) and MCPyV–negative (orange) patients with MPNs. Vertical lines represent the interquartile range, and white dots indicate the mean age (67.5 years). Statistical comparisons: age vs. MCPyV (*t* test, *P* = 0.9657) and sex vs. MCPyV (*χ*^2^, *P* = 0.0372). **B,** Bar chart showing the distribution of MPN subtypes in MCPyV-positive (blue) and –negative (orange) patients with MPNs. Statistical comparisons: ET vs. CML (Fisher exact test: *P* = 0.1513). *P* < 0.05 as statistical significance. F, female; M, male; MF, myelofibrosis; Neg, negative; Pos, positive; post-ET-MF, after ET myelofibrosis; post-PV-MF, after PV myelofibrosis.

MCPyV DNA was detected in 14 of 78 (17.9%) BM specimens. Among these, seven cases were positive with the LT3 primer set, eight with the VP1 primer set, and case ID 75 was positive for both. None of the cases were positive with the M1/M2 primer set (Fig. 1A and E; Supplementary Figs. S1–S3; Supplementary Table S3). In contrast, HPyV6 DNA was identified in only 2 of 78 (2.56%) specimens (Fig. 1B and E; Supplementary Fig. S4; Supplementary Table S3). Among the 14 positive cases, the presence of MCPyV DNA was significantly associated with female sex (*χ*^2^, *P* = 0.0327) but not with age (*t* test, *P* = 0.9657; Table 1; Fig. 2A).

Of note, the statistical analyses demonstrated a significant association between MCPyV and MPNs compared with the prevalence of HPyV6 DNA (Fisher exact test, *P* = 0.0026; Fig. 1E). In detail, of the 78 MPN DNAs tested, MCPyV DNA was detected in 14.1% of ET (*n* = 11), 1.28% of PMF (*n* = 1), 1.28% of CML (*n* = 1), and 1.28% of post-ET-MF (*n* = 1). However, no statistically significant association between MCPyV positivity and the various types of MPNs was observed (Table 1; Fig. 2B). In addition, no statistically significant association was observed between MCPyV positivity and most genetic mutations in the MPNs (Table 1; Supplementary Fig. S5A), nor was there a significant difference in peripheral blood counts between MCPyV-positive and -negative cases (Table 1; Supplementary Fig. S5B). However, the MPL mutation showed a statistically significant association with MCPyV-positive and -negative cases (Fisher exact test, *P* = 0.0243, Table 1).

Interestingly, in the control group of BM specimens from patients who underwent femoral head replacement surgery (*n* = 66), only 2/66 (3%) tested positive by MCPyV DNA PCR, and none of the patients tested positive for HPyV6 DNA (Fig. 1F; Supplementary Figs. S6–S9). By comparing the presence of MCPyV in MPNs with healthy control groups (BM), we found a statistically significant association for MCPyV DNA (Fisher exact test, *P* = 0.0063; OR = 7; 95% CI, 1.53–32.06; Fig. 1C; Table 2) but not for HPyV6 DNA (Fisher exact test, *P* = 0.5; OR = 4.35; 95% CI, 0.20–92.16; Fig. 1D; Table 2). In addition, we compared the prevalence of MCPyV DNA in healthy BM specimens with published data on MCPyV DNA prevalence in healthy blood donors (10–13). Interestingly, the MCPyV positive rate in healthy controls was significantly lower than that in whole blood (*χ*^2^, *P* = 0.00003) and buffy coat (*χ*^2^, *P* = 0.00317) but did not differ significantly from serum (*χ*^2^, *P* = 0.7843; Table 3).

**Table 2.** MCPyV and HPyV6 prevalence in patients with MPNs compared with healthy controls.

Virus	MPN positivity (*n*, %)	MPN negativity (*n*, %)	Control group positivity (*n*, %)	Control group negativity (*n*, %)	OR vs. control (95% CI)	*P*
MCPyV	14 (17.95%)	64 (82.05%)	2 (3.03%)	64 (96.97%)	7 (1.53–32.06)	0.0063^a^
HPyV6	2 (2.56%)	76 (97.44%)	0 (0%)	66 (100%)	4.35 (0.20–92.16)^b^	0.500^a^

a Fisher exact test; statistical critics <0.05.

b OR and 95% CIs were calculated using the Haldane–Anscombe correction, in which 0.5 was added to all zero-value cell.

**Table 3.** Comparison of MCPyV prevalence between BM with blood-derived specimens in healthy donors.

Specimens	Reference	MCPyV		Total	*P* value^a^
		Positive	Negative		
BM	Our health cohort	2	64	66	​
Whole blood	(12, 25)	73	187	260	0.00003^b^
Serum	(10, 13)	11	629	640	0.7843^b^
Buffy coats	(11)	13	47	60	0.00317^b^

a *χ* ^2^ test, statistical critics <0.05.

b Comparison with BM group.

**Figure 3.**
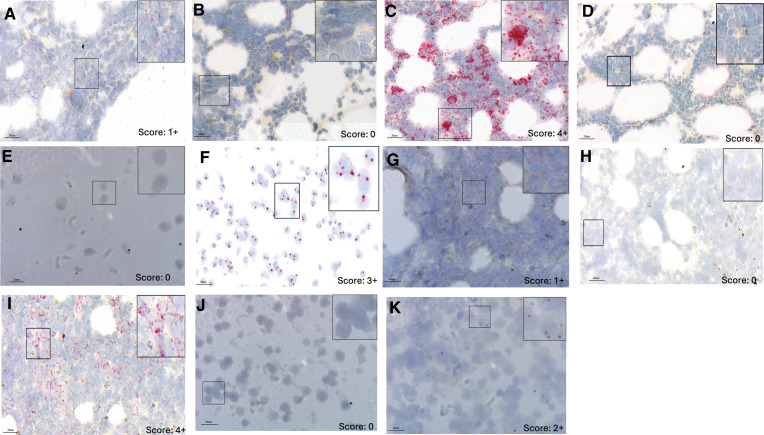
Detection of MCPyV and HPyV6 mRNA in formalin-fixed, paraffin-embedded MPN tissues and control cell lines by RNAscope. **A–C,** BM section from case ID47 hybridized with labeled MCPyV probes showing perinuclear and/or nuclear dots indicative of MCPyV transcripts (**A**, score 1+). The bacterial gene (DapB) served as a negative control (**B**, score 0), and the housekeeping gene (PPIB) as a positive control (**C**, score 4+) confirmed assay quality. **D–F,** ID82 hybridized with labeled MCPyV showed no detectable transcript signal (**D**, score 0). MCPyV-negative cell line MCC26 was used as a negative control and showed no specific signal (**E**, score 0), and the MCPyV-positive cell line WaGa served as a positive control, showing distinct red signal dots (**F** score 3+). **G–I,** BM section from case ID114 hybridized with labeled HPyV6 probes showing HPyV6 mRNA transcripts (**G**, score 1+). The DapB probe negative control (**H**, score 0) and the PPIB positive control (**I**, score 4+) confirmed assay quality. **J** and **K,** HEK293-empty vector cells served as a negative control (**J**, score 0), and HEK293-HPyV6ER cells as a positive control (**K**, score 2+). Images were acquired using 40× objective magnification via digital slide scanning. Insets show digitally zoomed-in regions for clarity. Score (0–4) indicate relative signal intensity, as described in the “Materials and Methods” section.

PCR-positive cases for MCPyV and HPyV6 were analyzed using RISH (Supplementary Table S3). Among 7 of the 14 MCPyV-positive MPN cases, MCPyV mRNA was detected as specific red punctate signals within the nucleus and/or cytoplasm, and all of them showed weak signals (score = 1+; Fig. 3A; Supplementary Table S3); of the remaining cases, three were negative (Fig. 3D), three yielded inconclusive results, and one was not tested due to sample unavailability. The MCPyV-positive MCC cell line WaGa and MCPyV-negative MCC cell line MCC26 served as the positive and negative controls, respectively (Fig. 3E and F). The two HPyV6-positive cases showed positive RISH signals (Fig. 3G). HEK293 cells transduced with a lentiviral vector expressing the HPyV6 early region T antigen were used as positive controls, whereas cells transduced with an empty vector were used as negative controls (Fig. 3J and K). Housekeeping gene human PPIB and bacterial DapB were used as the positive and negative controls, respectively (Fig. 3B, C, H, and I). Notably, ID64 exhibited expression of both MCPyV and HPyV6 transcripts.

**Figure 4.**
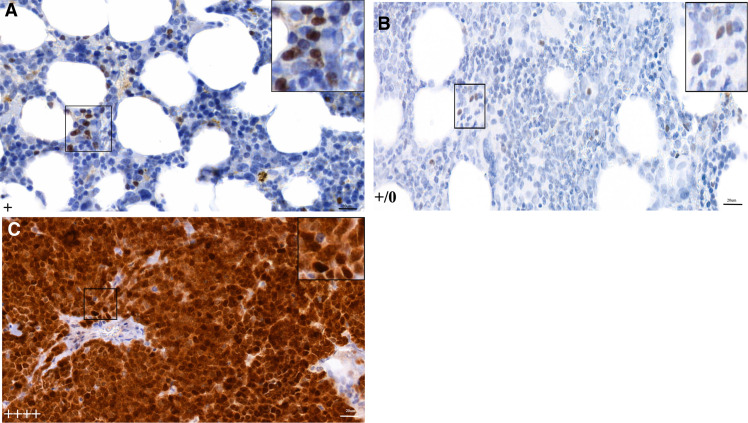
Representative images (40× magnification) showing MCPyV large T-antigen expression by IHC. **A,** BM tissue from case ID 47 from Zuyderland medical center showing weak (+) nuclear staining restricted to scattered lymphocytes. **B,** BN tissue from case ID 8 from (Maastricht UMC+) showing spare nuclear staining in lymphocytes only. **C,** MCC tissue used as positive control showing very strong (++++) nuclear staining of tumor cells. Brown nuclei indicate MCPyV large antigen expression.

IHC for the MCPyV large antigen was performed on PCR-positive MPN samples and three additional ET cases from the MUMC+ cohort. Positive nuclear staining was observed exclusively in scattered lymphocytes, whereas MPN were negative for large T antigen expression (Fig. 4; Supplementary Table S3).

## Discussion

Nonneoplastic (6, 11, 12) and neoplastic (8, 9) leukocytes have been identified as sites of MCPyV persistence. We hypothesized that other blood cells, including those of erythrocytic and megakaryocytic lineages, may also carry MCPyV. Interestingly, the megakaryocytic lineage in mice has been shown to be targeted by middle T gene expression in mouse polyomaviruses (16). These mice developed an acute myeloproliferative thrombocythemic disorder characterized by hyperplasia and proliferation of the megakaryocytic lineage. In addition, recent studies have shown that JAK inhibitors used for MPN treatment may increase the risk of MCC, potentially due to immunosuppression (14, 15). For example, ruxolitinib, a standard treatment for MPNs, has been linked to an increase in MCC.

In the present study, we examined freshly processed BM from patients with MPNs and compared them with BM obtained from femoral head replacements. MCPyV DNA was detected almost exclusively in MPN cases, whereas HPyV6 showed only sporadic positivity. Among the 14 MCPyV DNA–positive MPN cases, seven showed detectable MCPyV mRNA by RNAscope, typically corresponding to weak punctate signals. The overall low signal intensity and limited number of positive cells suggest low-level viral transcription. When combined with the IHC results showing expression restricted to lymphocytes rather than megakaryocytes or neoplastic cells, this pattern indicates that MCPyV may persist in peripheral blood lymphocytes in a latent or episomal state (7) and that oncogenic transformation requires integration and continuous expression of T antigens within tumor clones (3). The absence of large T antigens in MPN cells therefore suggests that MCPyV is unlikely to play a direct oncogenic role.

The finding that MCPyV DNA was mainly found in the BM of patients with ET is even more exciting. The above-mentioned findings of middle T-antigen expression in mice, which induced acute myeloproliferative thrombocythemic disorders in the BM, may indicate that polyomaviruses can affect the megakaryocytic lineage. Specifically, MCPyV may infect and affect the megakaryocytic lineage in humans. Although the low MCPyV DNA prevalence in our control BM cohort aligns with previously reported rates in serum (10, 13), it remains substantially lower than the levels described in whole blood and buffy coat (11, 12, 25). However, previously reported findings of MCPyV persistence in both nonneoplastic and neoplastic leukocytes may help explain the presence of MCPyV in megakaryocytes, particularly in the context of immunosuppression. To the best of our knowledge, this is the first study to examine freshly isolated BM DNA of MPN and controls, revealing a potential enrichment of MCPyV in BM.

Interestingly, the prevalence of MCPyV DNA in BM samples from patients with MPN was significantly higher than that in samples from healthy controls. However, no significant differences were found between MCPyV-positive and -negative MPN cases in terms of genetic mutations (JAK2 V617F, JAK2 exon 12, CARL, and BCR-ABL), peripheral blood counts, diagnosis subtypes, or patient age. This suggests that MCPyV is not directly involved in clonal expansion. The observed difference may be linked to immunosuppression due to hematologic diseases in MPNs (26). Although a notable association with MPL mutations was observed, the small sample size warrants cautious interpretation of this finding. Interestingly, whereas MCPyV-positive MCCs are more commonly reported in males (26), we observed a significantly higher incidence of MCPyV-positive MPNs in females.

Although we were excited about the detection of MCPyV DNA in the BM of ET patients, we acknowledge that this pilot study requires validation in larger cohorts. However, our findings require further validation in larger cohorts. It is tempting to speculate that the mere presence of MCPyV DNA is not proof of causal involvement or relationship between MCPyV and ET. In addition, this study did not assess viral integration or downstream functional consequences. Future investigations using long-read sequencing and integration site mapping in larger cohorts are necessary to clarify whether MCPyV can integrate into hematopoietic genomes and contribute to disease evolution. However, the detection of MCPyV DNA in MPN BM samples may offer new insights into the etiopathogenesis of a significant subset of MPNs.

## Supplementary Material

Figure S1 Figure S1. Detection of MCPyV DNA in the bone marrow of MPN patients using M1/M2 primers.

Figure S2 Figure S2. Detection of MCPyV DNA in the bone marrow of MPN patients using LT3 primers.

Figure S3 Figure S3. Detection of MCPyV DNA in the bone marrow of MPN patients using VPI primers.

Figure S4 Figure S4. Detection of HPyV6 DNA in the bone marrow of MPN patients using sTag primers.

Figure S5 Figure S5. Blood cell counts and genetic mutation profiles in MCPyV-positive and MCPyV-negative MPN cases.

Figure S6 Figure S6. Detection of MCPyV DNA in the bone marrow from healthy controls using M1/M2 primers.

Figure S7 Figure S7. Detection of MCPyV DNA in the bone marrow from healthy controls using LT3 primers.

Figure S8 Figure S8. Detection of MCPyV DNA in the bone marrow from healthy controls using VPI primers.

Figure S9 Figure S9. Detection of HPyV6 DNA in the bone marrow from healthy controls using sTag primers.

Table S1 Table S1. Distribution of Myeloproliferative Neoplasm Subtypes in this study

Table S2 Table S2. Demographics information of the study cohorts

Table S3 Table S3. Summary of positive PCR and RNA in situ hybridization results of MCPyV and HPyV6

## Data Availability

The data generated in this study are included in this article and its Supplementary Materials. Additional data are available upon request. Due to patient privacy considerations, individual clinical data are not publicly available.
