# Supplementary figures and images for: Predominant Merkel Cell Polyomavirus DNA Detection in Essential Thrombocythemia within Myeloproliferative Neoplasms
Source: Cancer Res Commun. 2026 Apr 3;6(4):742–9. doi: 10.1158/2767-9764.CRC-25-0471 (PMC13047360; doi:10.1158/2767-9764.CRC-25-0471)

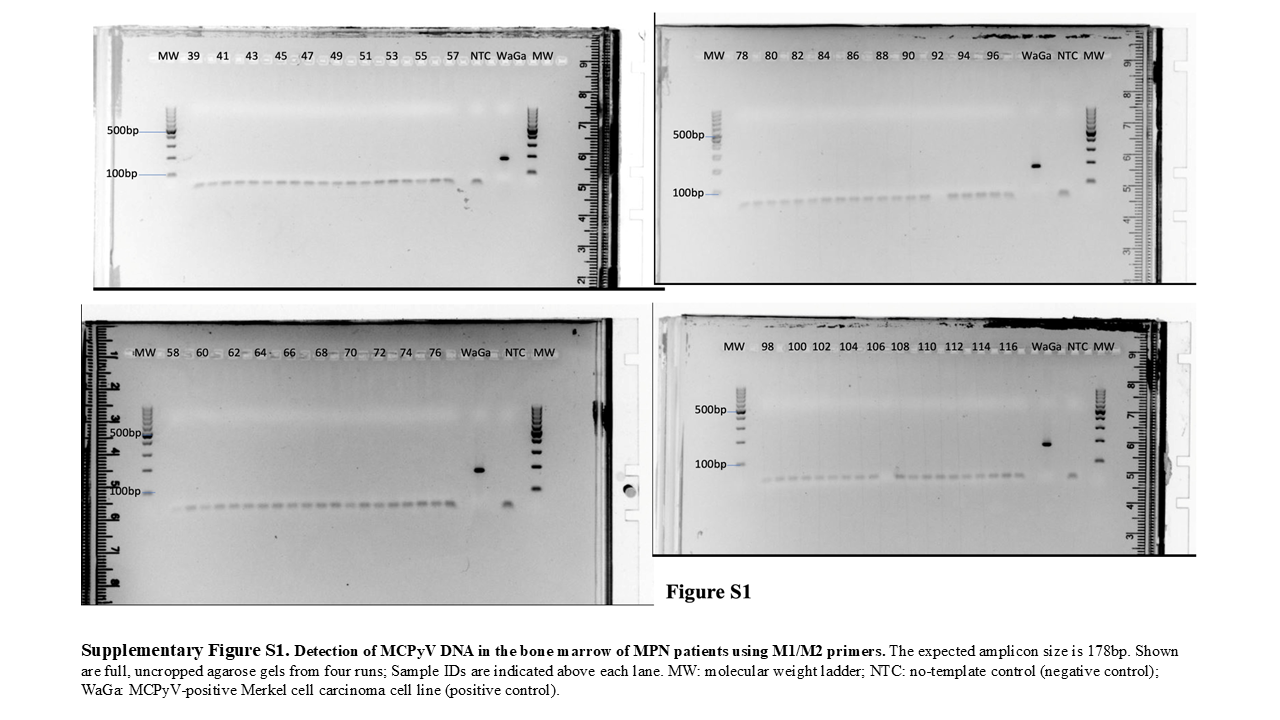

Supplement: Figure S1 — Detection of MCPyV DNA in the bone marrow of MPN patients using M1/M2 primers. [file crc-25-0471_figure_s1_suppsf1.png]

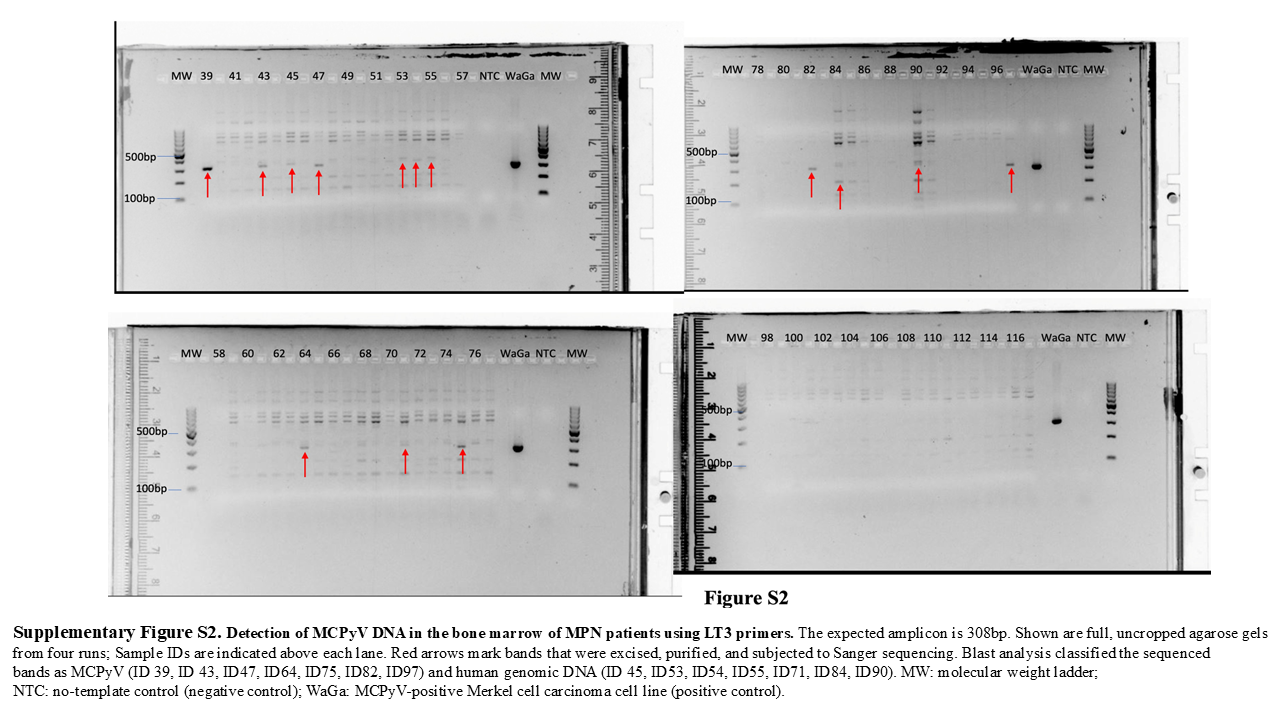

Supplement: Figure S2 — Detection of MCPyV DNA in the bone marrow of MPN patients using LT3 primers. [file crc-25-0471_figure_s2_suppsf2.png]

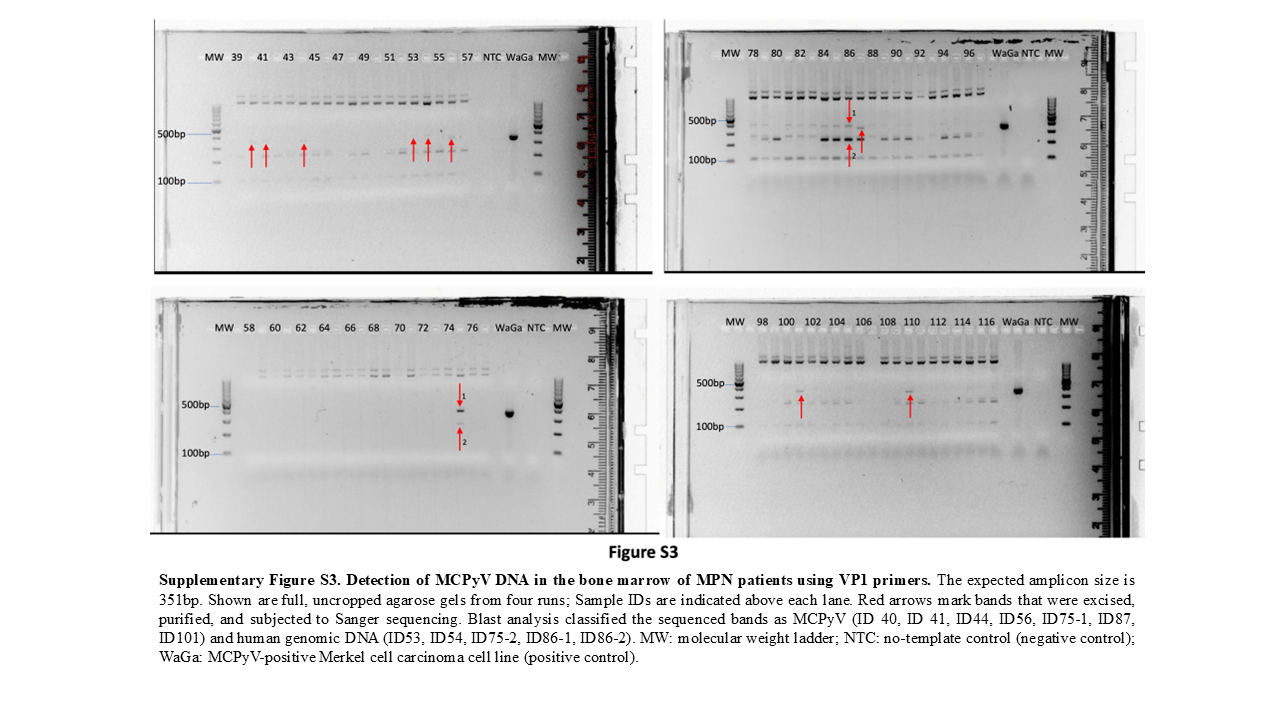

Supplement: Figure S3 — Detection of MCPyV DNA in the bone marrow of MPN patients using VPI primers. [file crc-25-0471_figure_s3_suppsf3.png]

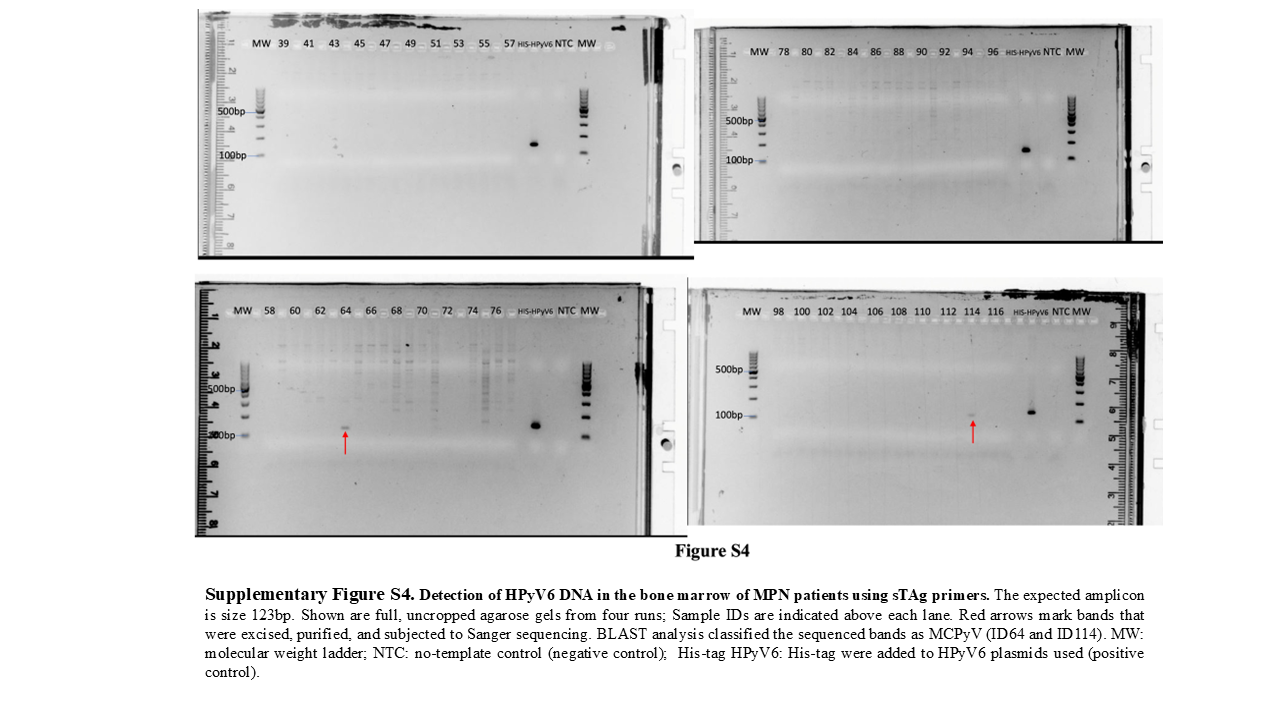

Supplement: Figure S4 — Detection of HPyV6 DNA in the bone marrow of MPN patients using sTag primers. [file crc-25-0471_figure_s4_suppsf4.png]

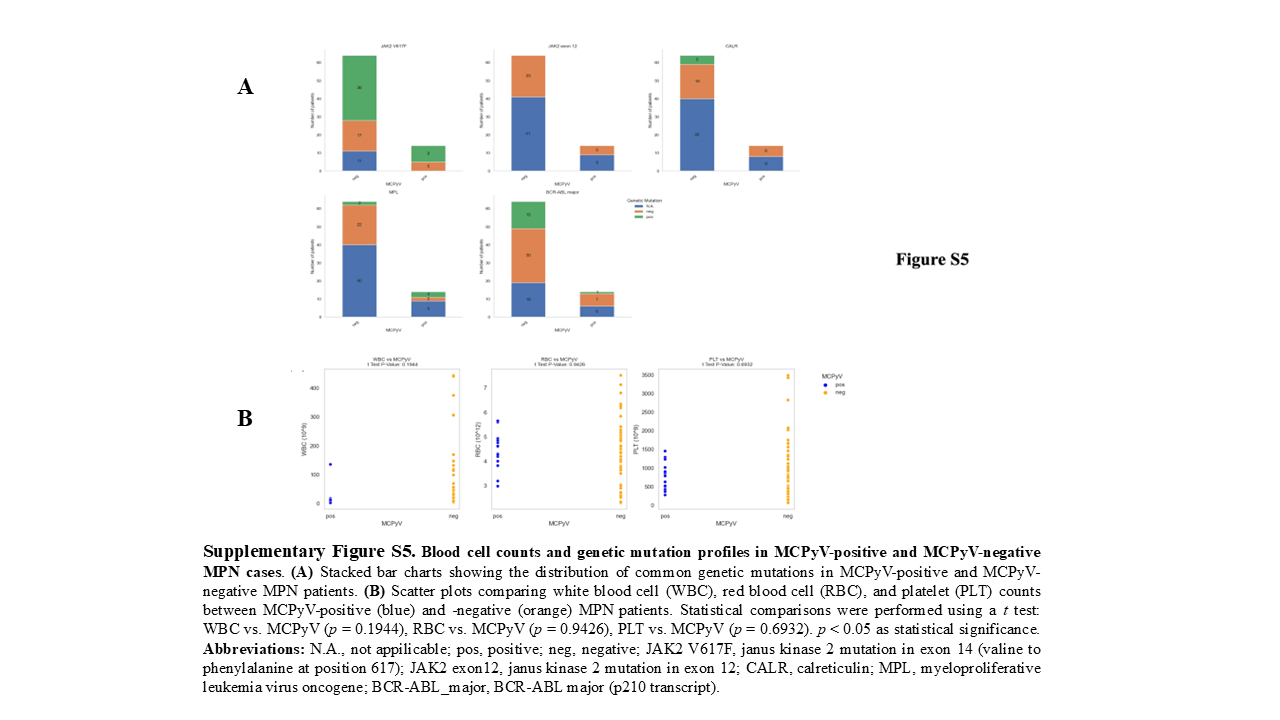

Supplement: Figure S5 — Blood cell counts and genetic mutation profiles in MCPyV-positive and MCPyV-negative MPN cases. [file crc-25-0471_figure_s5_suppsf5.png]

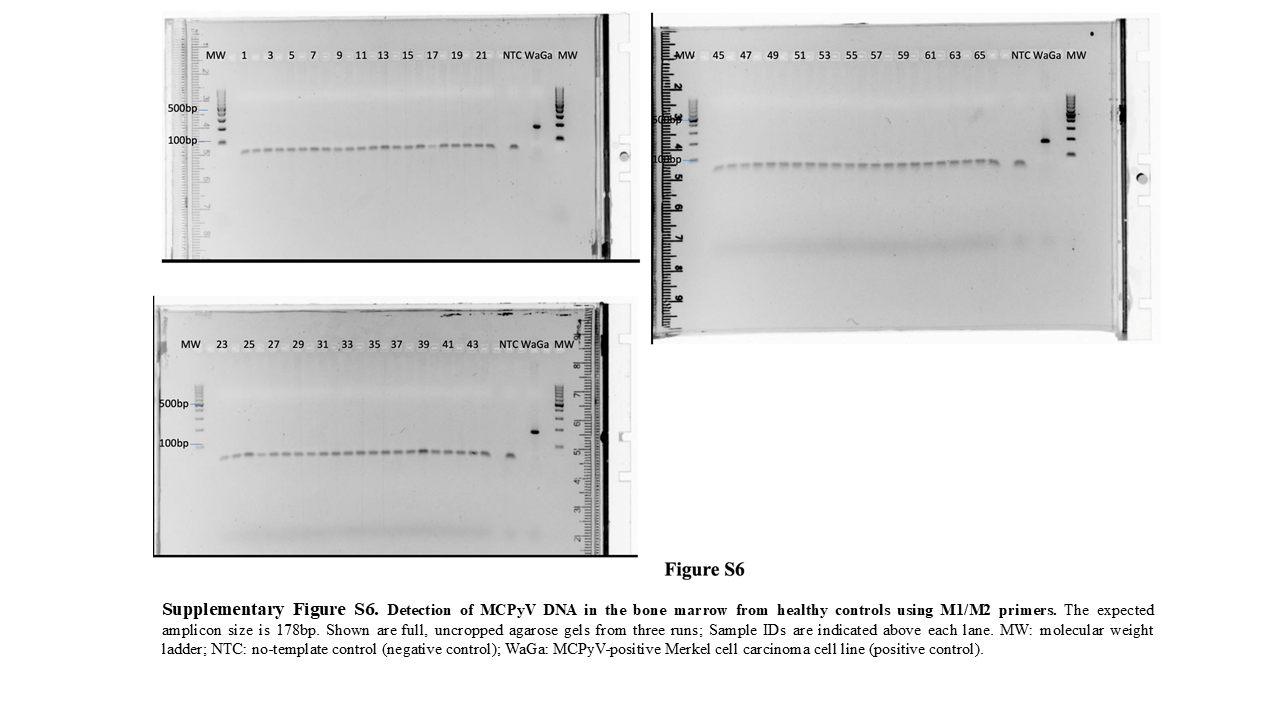

Supplement: Figure S6 — Detection of MCPyV DNA in the bone marrow from healthy controls using M1/M2 primers. [file crc-25-0471_figure_s6_suppsf6.png]

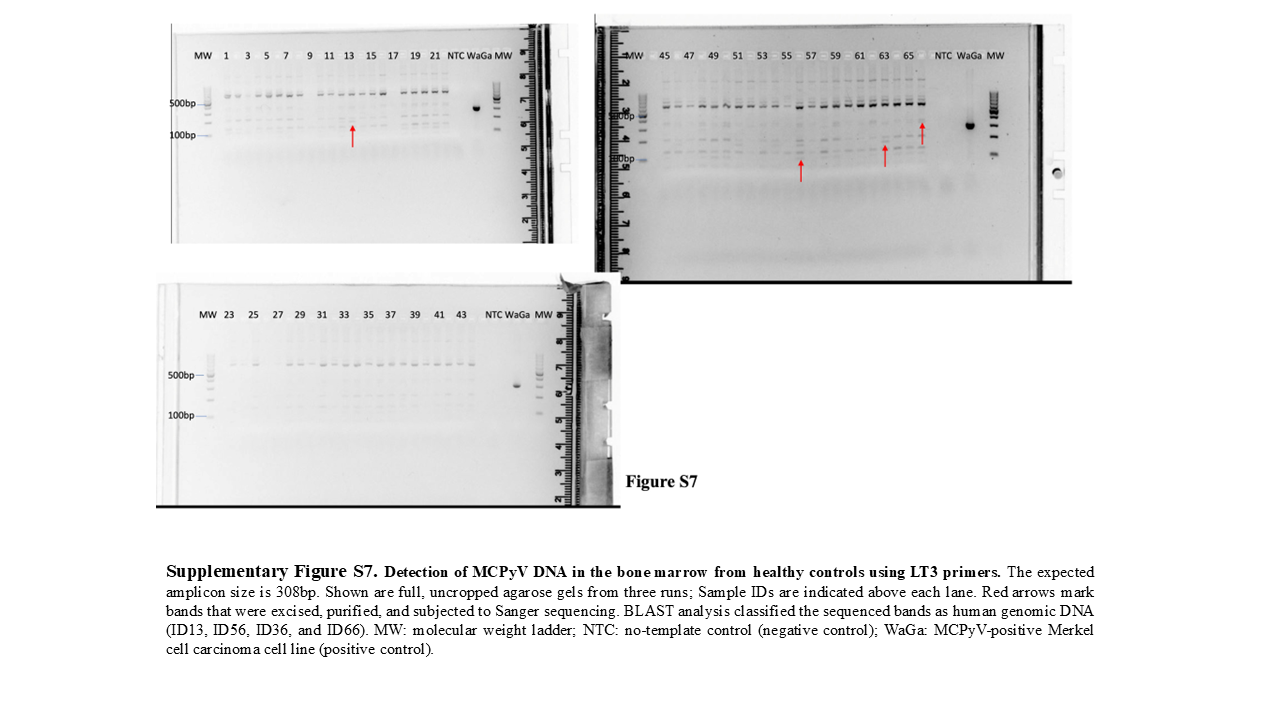

Supplement: Figure S7 — Detection of MCPyV DNA in the bone marrow from healthy controls using LT3 primers. [file crc-25-0471_figure_s7_suppsf7.png]

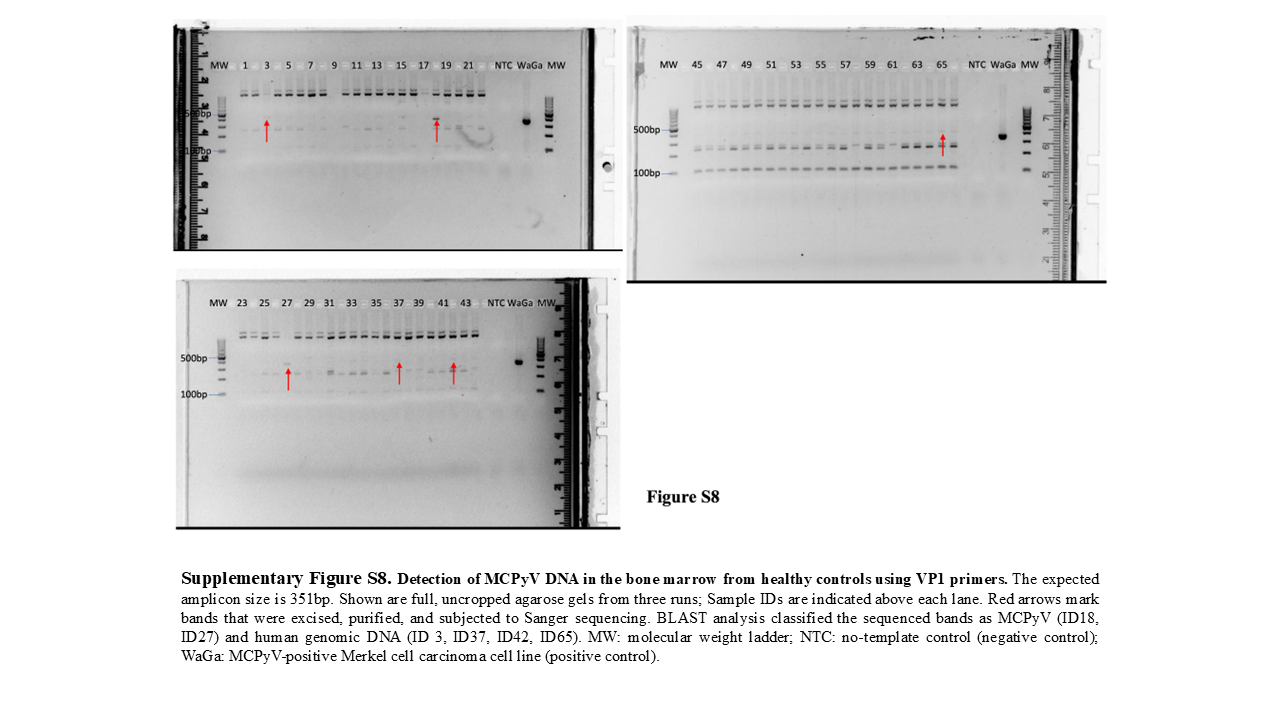

Supplement: Figure S8 — Detection of MCPyV DNA in the bone marrow from healthy controls using VPI primers. [file crc-25-0471_figure_s8_suppsf8.png]

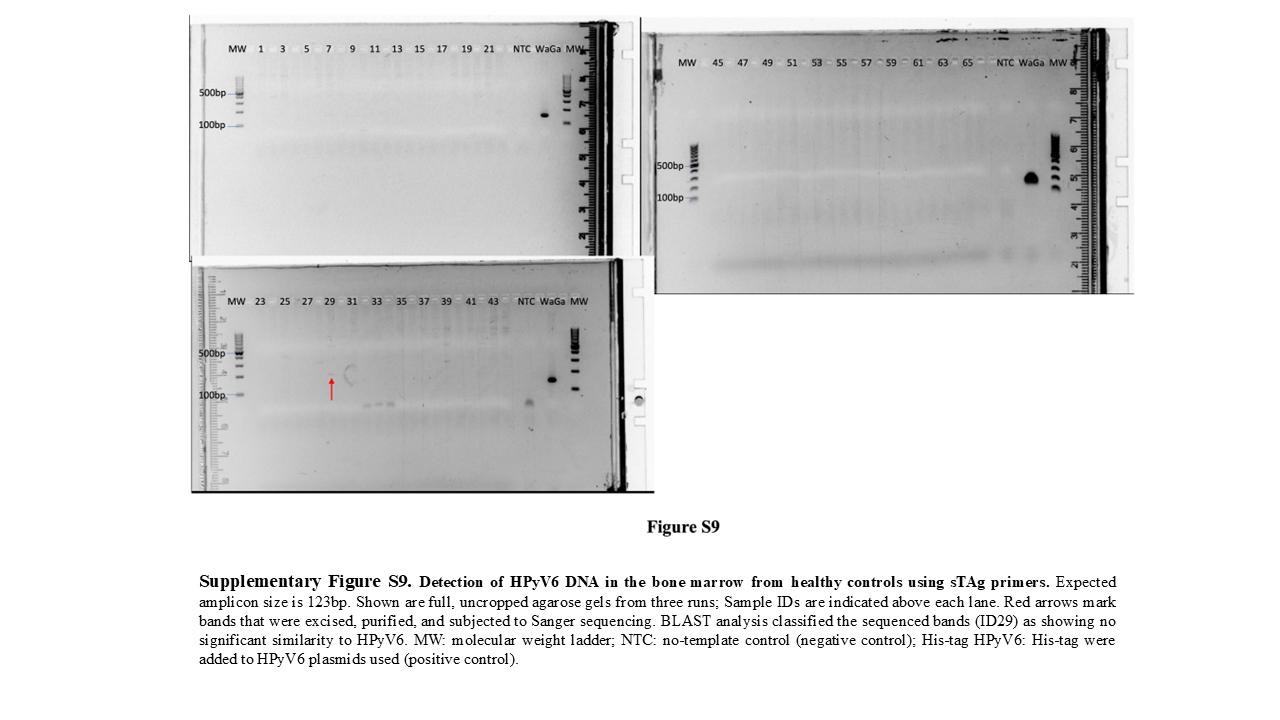

Supplement: Figure S9 — Detection of HPyV6 DNA in the bone marrow from healthy controls using sTag primers. [file crc-25-0471_figure_s9_suppsf9.png]
