# Supplementary material for: Predominant Merkel Cell Polyomavirus DNA Detection in Essential Thrombocythemia within Myeloproliferative Neoplasms
Source: Cancer Res Commun. 2026 Apr 3;6(4):742–9. doi: 10.1158/2767-9764.CRC-25-0471 (PMC13047360; doi:10.1158/2767-9764.CRC-25-0471)
Supplement: Table S1 — Distribution of Myeloproliferative Neoplasm Subtypes in this study [file crc-25-0471_table_s1_suppst1.docx]

**Supplementary Table S1. Distribution of Myeloproliferative Neoplasm Subtypes in this study Cohort**

| Diagnosis | Number of Patients (n) | Percentage (%) |
| --- | --- | --- |
| Essential Thrombocythemia (ET) | 44 | 56.4% |
| Polycythemia Vera (PV) | 6 | 7.7% |
| Primary Myelofibrosis (PMF) | 3 | 3.85% |
| Post-ET Myelofibrosis (post-ET-MF) | 5 | 6.4% |
| Post-PV Myelofibrosis (post-PV-MF) | 3 | 3.85% |
| Chronic Myeloid Leukemia (CML) | 17 | 21.8% |
