# Supplementary material for: Predominant Merkel Cell Polyomavirus DNA Detection in Essential Thrombocythemia within Myeloproliferative Neoplasms
Source: Cancer Res Commun. 2026 Apr 3;6(4):742–9. doi: 10.1158/2767-9764.CRC-25-0471 (PMC13047360; doi:10.1158/2767-9764.CRC-25-0471)
Supplement: Table S2 — Demographics information of the study cohorts [file crc-25-0471_table_s2_suppst2.docx]

| **Cohort** | ***n*** | **Age, years (median [range])** | **Male, n (%)** | **Female, n (%)** | **Specimen source** |
| --- | --- | --- | --- | --- | --- |
| **MPN** | 78 | 67.5 [29-87] | 45 (57.7%) | 33 (42.3%) | Bone Marrow |
| **Controls** | 66 | NA | NA | NA | Bone marrow (femoral head surgery) |

**Supplementary Table S2. Demographics information of the study cohorts**

NA: not available
