# Supplementary material for: Predominant Merkel Cell Polyomavirus DNA Detection in Essential Thrombocythemia within Myeloproliferative Neoplasms
Source: Cancer Res Commun. 2026 Apr 3;6(4):742–9. doi: 10.1158/2767-9764.CRC-25-0471 (PMC13047360; doi:10.1158/2767-9764.CRC-25-0471)
Supplement: Table S3 — Summary of positive PCR and RNA in situ hybridization results of MCPyV and HPyV6 [file crc-25-0471_table_s3_suppst3.docx]

**Supplementary Table S3.**

**Summary of positive PCR and RNA in situ hybridization results of MCPyV and HPyV6**

| **LabID** | **Diagnosis** | **MCPyV (PCR)** | | | **IHC (CM2B4)** | **RISH (MCPyV)** | **HPyV6(PCR)** | **RISH**  **(HPyV6)** |
| --- | --- | --- | --- | --- | --- | --- | --- | --- |
|  |  | **M1/M2** | **LT3** | **VP1** |  |  |  |  |
| 39 | ET | - | + | - | lymphocyte | 1+ | - | N.D. |
| 40 | ET | - | - | + | lymphocyte | 1+ | - | N.D. |
| 41 | ET | - | - | + | lymphocyte | Inconclusive | - | N.D. |
| 43 | CML | - | + | - | lymphocyte | Inconclusive | - | N.D. |
| 44 | ET | - | - | + | lymphocyte | 1+ | - | N.D. |
| 47 | ET | - | + | - | lymphocyte | 1+ | - | N.D. |
| 56 | ET | - | - | + | lymphocyte | - | - | N.D. |
| 64 | PMF | - | + | - | lymphocyte | 1+ | + | 1+ |
| 75 | ET | - | + | + | lymphocyte | - | - | N.D. |
| 82 | ET | - | + | - | lymphocyte | - | - | N.D. |
| 87 | ET | - | - | + | N.D. | N.D. | - | N.D. |
| 97 | post-ET-MF | - | + | - | lymphocytes | 1+ | - | N.D. |
| 101 | ET | - | - | + | lymphocyte | Inconclusive | - | N.D. |
| 110 | ET | - | - | + | lymphocyte | 1+ | - | N.D. |
| 114 | PV | - | - | - | N.D. | - | + | 1+ |
| 6 | ET | - | - | - | Inconclusive | N.D. | N.D. | N.D. |
| 7 | ET | - | - | - | Inconclusive | N.D. | N.D. | N.D. |
| 8 | ET | - | - | - | Inconclusive | N.D. | N.D. | N.D. |

**Abbreviation:** ET, essential thrombocythemia; PV, polycythemia vera; PMF, primary myelofibrosis; post-ET-MF, post-ET myelofibrosis; N.D., not done; +, positive; -, negative; 1+, 1-3 dots per positive cell.
